# Predictors of functional deterioration from 90 days to 1 year after endovascular treatment for vertebrobasilar artery occlusion: a multicenter retrospective study

**DOI:** 10.3389/fneur.2025.1671672

**Published:** 2025-11-03

**Authors:** Lei Wang, Wen Sun, Lei Ping, Guofang Chen, Xinmin Fu, Leijing Liu, Qiao Li, Meixue Yao

**Affiliations:** ^1^Department of Neurology, Xuzhou Central Hospital, Xuzhou, China; ^2^Department of Neurology, Xuzhou Clinical School of Xuzhou Medical University, Xuzhou, China; ^3^Department of Neurology Centre for Leading Medicine and Advanced Technologies of IHM, The First Affiliated Hospital of USTC Division of Life Sciences and Medicine, University of Science and Technology of China, Hefei, China; ^4^Center for Medical Statistics and Data Analysis, School of Public Health, Xuzhou Medical University, Xuzhou, China

**Keywords:** stroke, vertebrobasilar artery occlusion, endovascular treatment, long-term prognosis, risk factors

## Abstract

**Objective:**

To investigate risk factors for delayed neurological deterioration (1-year mRS score > 90-day score) in patients with acute ischemic stroke due to vertebrobasilar artery occlusion (VBAO) after endovascular treatment (EVT), and to evaluate heterogeneity across age and sex subgroups.

**Methods:**

This multicenter retrospective study included 1,342 VBAO patients who underwent EVT between December 2015 and June 2022. Based on dynamic changes in the mRS score, patients were categorized into a 1-year functional deterioration group (*n* = 288) and a functional stability group (*n* = 1,054). Multivariate binary logistic regression was used to identify factors associated with long-term functional deterioration. Stratified analyses were further performed by age (≤70 years vs. >70 years) and sex.

**Results:**

After adjusting for demographics and clinical variables, the following factors were independently associated with 1-year functional deterioration (all *p* < 0.05): female sex (OR = 1.425, 95%CI:1.062–1.911), increasing age (per year) (OR = 1.016, 95%CI: 1.004–1.028), Door-to-Puncture Time (DTP) (OR = 1.001, 95%CI: 1.000–1.001), modified Thrombolysis in Cerebral Infarction (mTICI) score (2b, OR = 0.452, 95%CI: 0.234–0.874; 3, OR = 0.398, 95%CI: 0.213–0.742) and number of attempts(≥4, OR = 3.765, 95%CI: 1.601–8.854). Gender-stratified analysis shows age and DTP drive female outcomes; male outcomes are regulated by complex surgical factors. Age stratification reveals younger females have poorer improvement than males with equal reperfusion, while in older patients, operation count strongly impacts outcomes, with high-quality reperfusion buffering multiple operations’ harm.

**Conclusion:**

This study revealed that the functional outcomes of patients with VBAO from 90 days to 1 year after EVT exhibit distinct patterns, with the dominant influencing factors varying by gender and age group. This provides an important basis for the formulation of individualized treatment strategies.

## 1 Introduction

VBAO represents the most lethal subtype of acute posterior circulation stroke. Although it accounts for only approximately 1% of all ischemic strokes and 5–10% of large vessel occlusion strokes (1, 2), it carries a devastatingly high mortality rate of 40–60% (3–5). Even when patients survive, they frequently suffer severe disability, significantly diminishing quality of life and imposing substantial burdens on both families and society. Advances in EVT technology, evolving from traditional intra-arterial thrombolysis to the widespread adoption of second-generation stent retrievers and aspiration systems, have significantly improved recanalization rates for VBAO. Data indicate that EVT enables 35–45% of patients to achieve functional independence [defined as a modified Rankin Scale (mRS) score of 0–3] at 90 days post-procedure, highlighting its substantial advantage in early intervention.

However, clinical follow-up reveals a critical concern: 20–30% of patients experience neurological deterioration beyond 90 days, and the 1-year mortality rate surges by 10–15% compared to the 90-day mark. This delayed functional decline involves complex, multifactorial mechanisms, including secondary brain injury stemming from reperfusion injury, chronic neurodegenerative changes, and the persistent progression of comorbidities such as hypertension and diabetes mellitus. Critically, the mechanisms and influencing factors governing functional changes during the pivotal post-procedural window from 90 days to 1 year remain systematically unexplored. This knowledge gap leaves clinicians without reliable evidence to guide long-term management strategies.

Existing research predominantly focuses on predictors of early (90-day) outcomes, such as the Posterior Circulation Alberta Stroke Program Early Computed Tomography Score (PC-ASPECTS), patient comorbidities (e.g., glycemic control, blood pressure), and procedural factors (e.g., puncture-to-recanalization time) (6–9). Addressing this crucial void, this multicenter cohort study aims to: (1) Identify independent predictors of functional deterioration (defined as an increase in mRS score ≥1 point) occurring between 90 days and 1 year following EVT, pinpointing key risk determinants; (2) Investigate the impact of age and sex on functional outcomes during this period, thereby uncovering potential population heterogeneity. The findings of this study are expected to provide robust evidence-based support for developing precise prognostic models and formulating individualized secondary prevention strategies. This research will fill a critical gap in understanding the mid-term (90-day to 1-year) functional trajectory of VBAO patients, ultimately enhancing clinical management strategies.

## 2 Methods

### 2.1 Study population

Data for this multicenter retrospective study were extracted from patients treated at 65 centers across 15 Chinese provinces between December 2015 and June 2022, following previously described methodologies (10, 11). Inclusion criteria were: (a) Age ≥18 years; (b) Confirmed diagnosis of VBAO by CT angiography, magnetic resonance angiography, or digital subtraction angiography; (c) Time from last known well to hospital presentation ≤24 h. Exclusion criteria comprised: (a) Pre-stroke mRS score >2; (b) Participation in any clinical trial; (c) Evidence of concurrent anterior circulation stroke, arteriovenous malformation, or intracranial aneurysm; (d) Pregnancy or lactation; (e) Incomplete critical baseline data or imaging studies. This study received approval from the Ethics Committee of The First Affiliated Hospital of University of Science and Technology of China (Approval No.: 2020–40). As a retrospective study, informed consent from patients or their legal representatives was waived.

### 2.2 Treatment protocol

EVT procedures included thrombectomy via aspiration, stent retriever thrombectomy, balloon angioplasty, intra-arterial thrombolysis, intracranial stenting, or any combination thereof, as determined by the treating physician.

### 2.3 Data collection

Comprehensive patient data were collected, encompassing demographics, clinical assessments [including the National Institutes of Health Stroke Scale (NIHSS) score], laboratory findings, neuroimaging results, treatment modalities, and complications. Early ischemic changes were quantified using the PC-ASPECTS. VBAO was classified as proximal (vertebrobasilar junction to mid-basilar artery), mid-basilar, or distal (mid-basilar artery to posterior cerebral artery origins). Additional parameters included procedure time(PT), DTP, number of thrombectomy attempts (No. of attempts), and post-procedural recanalization success (mTICI grade 2b-3). Specific details can be found in Appendix S1.

### 2.4 Follow-up

Excluding patients deceased within 90 days (typically with extreme baseline severity) permitted 1-year mRS evaluation without affecting baseline comparability between functional decline and stable groups. Functional outcomes were assessed using mRS at 90 days and 1 year post-EVT. Patients were stratified into two groups: the *functional deterioration group* (1-year mRS > 90-day mRS) and the *stable/improved group* (1-year mRS ≤ 90-day mRS).

### 2.5 Statistical analysis

In this study, three variables (stroke history, diastolic and systolic blood pressure) contained missing values (<1%), which were excluded from analysis given minimal absence rates. Continuous variables are presented as mean ± standard deviation (SD; normally distributed data) or median with interquartile range (IQR; non-normally distributed data), analyzed using the Mann–Whitney *U* test (non-parametric) or Student’s t-test (parametric). Categorical variables are expressed as frequencies (percentages), compared via Pearson’s chi-square or Fisher’s exact test. Binary logistic regression (adjusted for potential confounders) identified independent predictors of 1-year functional deterioration, with results reported as odds ratios (ORs) and 95% confidence intervals (95% CIs). Subgroup analyses were stratified by age (<70 vs. ≥70 years) and sex (female vs. male). To address the inherent bias in retrospective analyses, we have implemented specific mitigation strategies (detailed in Appendix S1). Analyses were performed using SPSS 26.0, and figures were generated with GraphPad Prism 5. A two-tailed *p* < 0.05 defined statistical significance.

## 3 Results

### 3.1 Baseline characteristics

**Figure 1:**
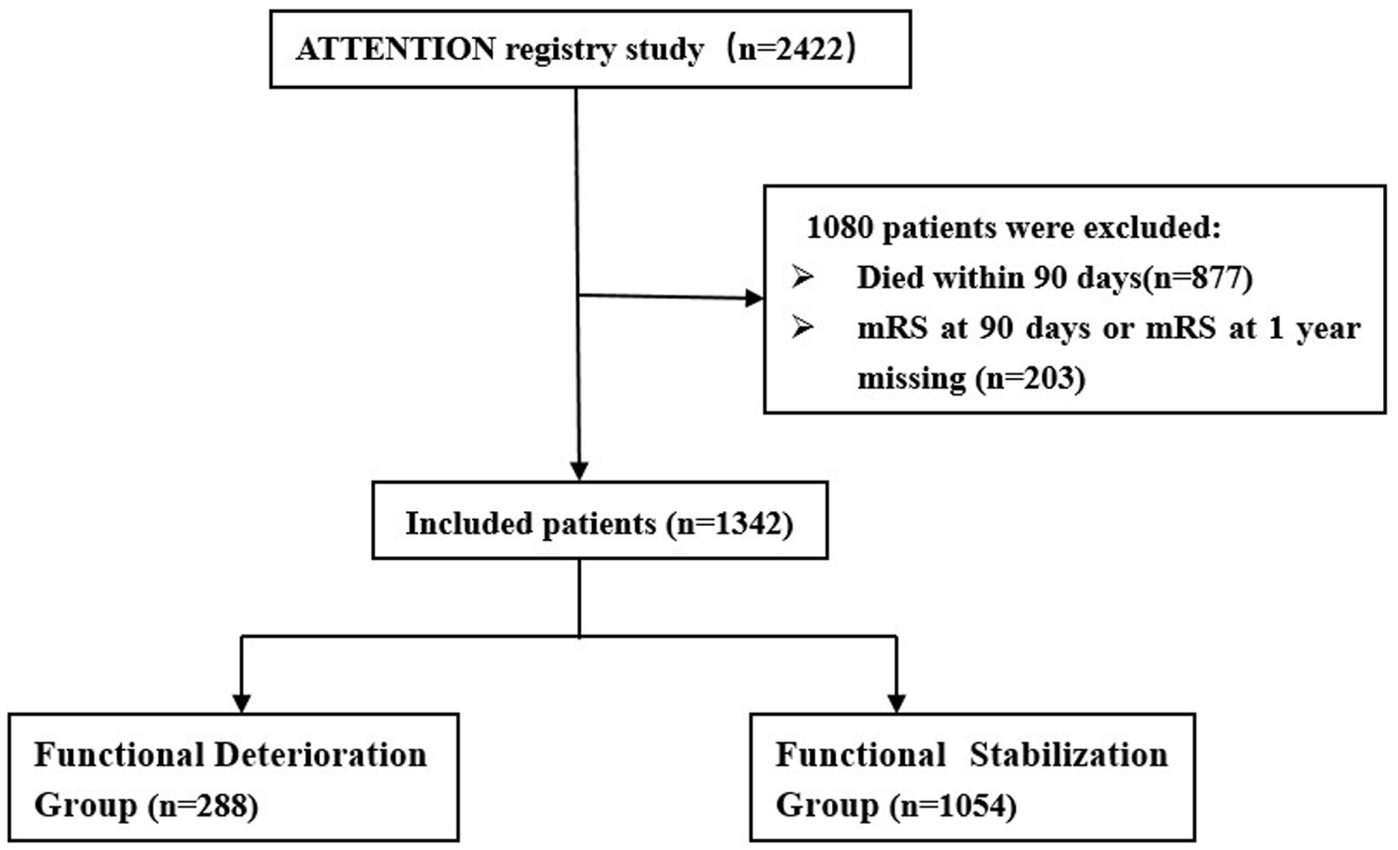
Flow diagram of the patient selection process.

**Figure 2:**
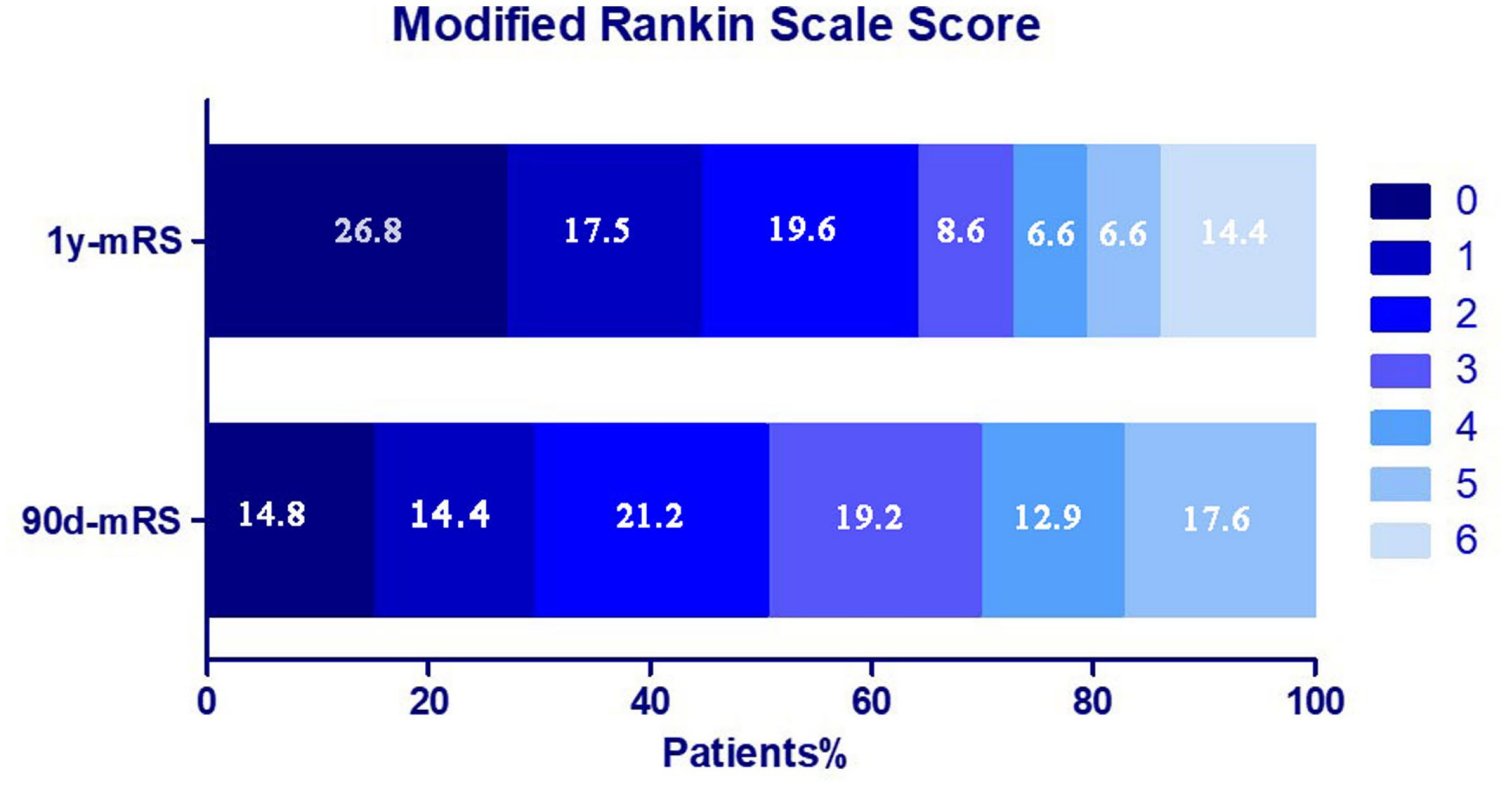
Distribution of the mRS score at 90 days and 1 year in two groups.

Among 2,422 VBAO patients in this retrospective study who underwent EVT, 877 patients were excluded due to death within 90 days, and 203 patients were excluded due to missing 90-day mRS or 1-year mRS data. Ultimately, 1,342 patients were included (288 in the 1-year functional deterioration group, 1,054 in the stable/improved group) (Figure 1). The distribution of mRS scores at 90 days and 1 year is shown in Figure 2. Baseline characteristics of the two groups are summarized in Table 1. Compared to the stable group, the deterioration group exhibited a lower proportion of males [190 (66.0%) vs. 789 (74.9%); *p* = 0.003], a higher median age [66.5 (IQR: 59–74) vs. 64 (IQR: 55–71); *p* = 0.002], lower median diastolic blood pressure [82 (IQR: 78–91) vs. 85 (IQR: 77–95); *p* = 0.007], lower admission glucose [6.4 (IQR: 5.7–8.1) vs. 6.8 (IQR: 5.9–8.5); *p* = 0.010], lower median PC-ASPECTS [8 (IQR: 7–10) vs. 9 (IQR: 8–10); *p* = 0.018]. Additionally, the distributions of the No. of attempts (*p* = 0.021) and mTICI grade (*p* = 0.002) differed significantly between the two groups. No significant differences were observed in other baseline variables (details in Table 1).

**Table 1:** Baseline characteristics of patients by the changes in functional outcomes.

Variable	Stabilization group (*n* = 1,054)	Deterioration group (*n* = 288)	*p* value
Age, median (IQR), years	64 (55,71)	66.5 (59,74)	0.002
Male, *n* (%)	789 (74.9)	190 (66.0)	0.003
Smoking, *n* (%)	353 (33.5)	105 (36.5)	0.347
Drinking, *n* (%)	236 (22.4)	69 (24.0)	0.574
Blood pressure on admission, mm Hg			
Systolic blood pressure, median (IQR)	145 (130, 162)	146 (132, 163)	0.576
Diastolic blood pressure, median (IQR)	85 (77,95)	82 (78,91)	0.007
Admission Blood Glucose	6.8 (5.9, 8.5)	6.4 (5.7, 8.1)	0.010
Triglyceride	1.35 (0.96,1.88)	1.41 (0.95, 2.03)	0.311
PT, median (IQR), min	105 (70, 140)	110 (75, 146)	0.122
DPT, median (IQR), min	300 (219, 460)	341 (210, 525)	0.173
Medical history, *n* (%)			
Atrial fibrillation, *n* (%)	211 (20.0)	70 (24.3)	0.113
Coronary heart disease, *n* (%)	106 (10.1)	33 (11.5)	0.489
Stroke or TIA, *n* (%)	222 (21.1)	67 (23.3)	0.404
Baseline NIHSS score, median (IQR)	20 (10,28)	20 (10,30)	0.154
Location of occlusion, *n* (%)			0.212
Proximal basilar-artery	273 (25.9)	77 (26.7)	
Middle basilar-artery	242 (23.0)	71 (24.7)	
Distal basilar-artery	203 (19.3)	40 (13.9)	
Vertebral artery V4	336 (31.9)	100 (34.7)	
TOAST, *n* (%)			0.350
LAA	689 (65.4)	191 (66.3)	
CE	200 (19.0)	61 (21.2)	
Other	165 (15.7)	36 (12.5)	
General anesthesia, *n* (%)	415 (39.4)	123 (42.7)	0.415
pc-ASPECTS, median (IQR)	9 (8, 10)	8 (7, 10)	0.018
sICH, *n* (%)	49(4.6)	14(4.9)	0.880
No. of attempts, *n* (%)			0.021
0	168 (15.9)	38 (13.2)	
1	507 (48.1)	127 (44.1)	
2	273 (25.9)	81 (28.1)	
3	91 (8.6)	30 (10.4)	
≥4	15 (1.4)	12 (4.2)	
mTICI grade, *n* (%)			0.002
0	32 (3.0)	18 (6.3)	
1	27 (2.6)	12 (4.2)	
2a	30 (2.8)	17 (5.9)	
2b	266 (25.2)	73 (25.3)	
3	699 (66.3)	168 (58.3)	

TIA, transient ischemic attack; LAA, large artery atherosclerosis; CE, cardioembolism; sICH, symptomatic intracranial hemorrhage.

### 3.2 Multivariate regression analysis

Based on univariate analysis and clinical experience, variables including age, sex, blood pressure, admission glucose, PC-ASPECTS, occlusion site, medical history, NIHSS, anesthesia type, PT, TOAST, DTP, No. of attempts, and mTICI grade were included in stepwise binary logistic regression. No multicollinearity was observed among variables [variance inflation factor (VIF) < 2], and the Hosmer-Lemeshow goodness-of-fit test confirmed satisfactory model calibration (*p* > 0.05). Detailed results are provided in Supplementary Tables S1 and S2.

The results demonstrated that for each 1-year increase in age, the risk of long-term functional deterioration post-procedure increased by 1.6% (OR = 1.016, 95% CI: 1.004–1.028, *p* = 0.007). Female patients had a significantly higher risk of functional deterioration at 1 year post-procedure compared to males, with an increased risk of 42.5% (OR = 1.425, 95% CI: 1.062–1.911, *p* = 0.018). Notably, each 1-min delay in DTP was linearly associated with a 0.1% increase in long-term functional deterioration risk (OR = 1.001, 95% CI: 1.000–1.002, *p* = 0.036). Patients requiring more than 3 thrombectomy attempts exhibited a more than tripled risk of 1-year functional deterioration (OR = 3.765, 95% CI: 1.601–8.854, *p* = 0.002). Furthermore, mTICI grade showed a significant inverse correlation with functional deterioration risk: patients achieving mTICI 2b (OR = 0.452, 95% CI: 0.234–0.874, *p* = 0.018) and mTICI 3 (OR = 0.398, 95% CI: 0.213–0.742, *p* = 0.004) exhibited 54.8 and 60.2% risk reductions, respectively. Detailed statistical results are presented in Table 2.

**Table 2:** Results of the multivariable logistic analysis.

Variable	Category	OR (95%CI)	*p* value
sex(male)		1.425 (1.062, 1.911)	0.018
age		1.016 (1.004, 1.028)	0.007
PC-ASPECTS		0.918 (0.842, 1.002)	0.054
DTP		1.001 (1.000,1.001)	0.036
No. of attempts^a^	1	1.220 (0.801, 1.857)	0.355
	2	1.441 (0.917, 2.266)	0.113
	3	1.419 (0.805, 2.501)	0.227
	‌ ≥ 4	3.765 (1.601, 8.854)	0.002
mTICI grade^b^	1	0.664 (0.263, 1.674)	0.385
	2a	0.945 (0.403, 2.216)	0.896
	2b	0.452 (0.234, 0.874)	0.018
	3	0.398 (0.213, 0.742)	0.004

The model included a probability of 0.1 and excluded a probability of 0.2.

a Using zero thrombectomy procedures as the reference.

b Using mTICI grade 0 as the reference.

### 3.3 Stratified analysis

Stratified analyses by gender and age were conducted in this study. For gender-stratified analyses, interaction terms including age × mTICI grade, age × No. of attempts, and mTICI grade × No. of attempts were incorporated. The Hosmer-Lemeshow goodness-of-fit test confirmed satisfactory model calibration (*p* > 0.05) (see Supplementary Table S3). Results showed that postoperative functional prognosis in female patients was primarily influenced by age (OR = 1.027, 95% CI: 1.005–1.050, *p* = 0.016) and DTP (OR = 1.001, 95% CI: 1.000–1.002, *p* = 0.005), with increases in both significantly elevating the risk of functional deterioration. In male patients, prognosis was affected by interaction effects of mTICI grade × No. of attempts (OR = 0.908, 95% CI: 0.829–0.995, *p* = 0.039) and age × No. of attempts (OR = 1.008, 95% CI: 1.004–1.013, *p* < 0.001).

The study population was divided into a younger group (aged <70 years) and an older group (aged ≥70 years). Age-stratified analysis incorporated interaction terms of sex × mTICI grade, sex × No. of attempts, and mTICI grade × No. of attempts. The regression equation had a good goodness-of-fit (*p* > 0.05) (see Supplementary Table S4). Results showed that in the younyer group, only the sex × mTICI grade interaction (OR = 1.173, 95% CI: 1.059–1.300, *p* = 0.002) influenced outcomes. In the older group, functional deterioration was associated with the No. of attempts, with risk increasing significantly with each additional attempt, and was further modulated by the mTICI grade × No. of attempts interaction (OR = 0.849, 95% CI: 0.753–0.956, *p* = 0.007). Detailed results are presented in Table 3.

**Table 3:** Results of stratified analysis by gender and age.

Grouping dimension	Category	Variable	OR (95%CI)	*p* value
Grouped by gender	Female	Age	1.027 (1.005, 1.050)	0.016
		PT	1.002 (0.999, 1.006)	0.114
		DTP	1.001 (1.000, 1.002)	0.005
		Age *No. of attempts	1.003 (0.999, 1.007)	0.098
	Male	pc-ASPECTS	0.910 (0.820, 1.010)	0.075
		mTICI grade* No. of attempts	0.908 (0.829, 0.995)	0.039
		Age *No. of attempts	1.008 (1.004, 1.013)	<0.001
		Age * mTICI grade	0.998 (0.996, 1.000)	0.085
Grouped by age	<70 years	pc-ASPECTS	0.899 (0.807, 1.001)	0.053
		Admission Blood Glucose	0.945 (0.886, 1.008)	0.088
		Sex* mTICI grade	1.173 (1.059, 1.300)	0.002
	≥70 years	DTP	1.001 (1.000, 1.002)	0.083
		No. of attempts (1)	1.174 (0.522, 2.644)	0.698
		No. of attempts (2)	4.111 (1.404, 2.644)	0.010
		No. of attempts (3)	7.288 (1.895, 28.033)	0.004
		No. of attempts (≥4)	37.189 (4.475, 309.064)	0.001
		mTICI grade* No. of attempts	0.849 (0.753, 0.956)	0.007

*Indicates an interaction term between variables.

## 4 Discussion

This study analyzed 1-year functional outcomes in 1342 VBAO patients after EVT. Each additional year increased functional deterioration risk by 1.9% [consistent with prior studies (12–14)], with younger patients showing better 1-year prognosis on Kaplan–Meier curves (15). Aging-related physiological decline (reduced vascular elasticity, weakened collaterals, comorbidities) impairs brain tolerance to ischemia–reperfusion injury. Older patients also have poorer recovery and higher rehabilitation complication rates, worsening outcomes.

Female VBAO patients had a 56.5% higher 1-year functional deterioration risk after EVT than males, contrasting with prior studies (15, 16) which found no sex differences or even better 90-day outcomes in females. These discrepancies likely stem from differing populations and follow-up durations (90-day vs. 1-year), indicating time-varying sex effects on prognosis. However, A meta-analysis (17) demonstrated significantly lower rates of favorable outcomes in females (OR 1.36, 95% CI 1.24 to 1.49, *p* < 0.00001). Consistent with this, a subsequent subgroup analysis of ischemic stroke patients showed that males maintained a greater likelihood of favorable outcomes at the one-year mark (OR 1.36, 95% CI 1.23 to 1.50, *p* < 0.0001), a finding that aligns with the results of our study. Estrogen’s neuroprotective and anti-inflammatory properties (18) benefit younger females acutely, but long-term effects are modulated by hormonal changes and sex-specific vascular traits. Gender differences in social roles, lifestyles, and disease management also indirectly affect recovery.

Although DTP did not differ significantly between groups in univariate analysis, its independent association with functional deterioration became statistically significant after adjusting for confounding variables including age, sex, and number of thrombectomy attempts. Consistent with the “time is brain” principle, our findings indicate that prolonged DTP elevates the risk of delayed functional decline. This relationship is particularly pronounced in posterior circulation ischemia (as seen in VBAO), where the brainstem and other critical structures exhibit poor tolerance to ischemia—with approximately 1.9 million neurons lost per minute of delay. Additionally, the limited collateral compensation capacity of the posterior circulation further amplifies the detrimental effects of delayed reperfusion, underscoring the time sensitivity of intervention in this patient population.

Surgical factors are pivotal in VBAO long-term prognosis. This study found >3 thrombectomy attempts raised functional deterioration risk, as repeated attempts may cause vascular injury, thrombus detachment, and distal embolism, exacerbating ischemia–reperfusion injury. Conversely, good postoperative mTICI (2b-3) reduced deterioration risk, highlighting successful revascularization’s key role. Bouslama et al. (19). (214 patients) showed revascularized VBAO patients had 10-fold higher good prognosis rates and halved mortality vs. non-revascularized. Clinically, improving first-attempt success and reducing unnecessary repeats may minimize complications and improve outcomes.

Gender-stratified analysis showed distinct prognostic patterns: females had “physiological vulnerability-dominant” features, with functional deterioration risk independently linked to age and prolonged DTP. This may relate to postmenopausal neurovascular unit degradation and estrogen attenuation (20), supporting a narrower therapeutic window in women with posterior circulation stroke (21). Males exhibited “technical interaction-dominant” patterns. Elderly men were sensitive to thrombectomy attempts (0.8% risk amplification/attempt, interaction OR = 1.008, *p* < 0.001), reflecting age-related vascular tolerance decline. Successful reperfusion (mTICI 2b/3) offset thrombectomy-related risks (interaction OR = 0.908, *p* = 0.039), emphasizing optimized recanalization for men’s long-term prognosis.

Age-stratified analysis showed that in the younger group, with the same reperfusion quality, females had significantly worse functional improvement than males. This may be related to the accelerated decline in collateral circulation compensation in elderly women, emphasizing the need for more active reperfusion strategies in this group. The prognosis of the elderly group is mainly determined by the number of operations: each additional thrombectomy attempt increases the risk of functional deterioration, highlighting the importance of the first pass effect (FPE) (22, 23). Notably, high-quality reperfusion can buffer the negative impact of multiple operations (mTICI × No. of attempts, OR = 0.849, *p* = 0.007), reducing the risk by 15.1%, suggesting that timely conversion of technical schemes is needed when the first attempt fails to achieve ideal reperfusion.

This study examines functional changes and determinants in VBAO patients 90 days to 1 year post-EVT. Unlike most research focusing on 90-day or 1-year outcomes, this critical transition period—vital for adjusting long-term rehabilitation strategies—takes center stage here. This study has several limitations. First, it adopted a retrospective design, which may introduce selection bias and thus undermine the reliability of causal inferences. Second, key data—including patients’ medication use, adherence to medical advice, post-discharge rehabilitation intensity, and the degree of rehabilitation standardization—during the 90-day to 1-year period after treatment may influence neurofunctional remodeling and long-term outcomes; however, this study failed to obtain such information. These findings represent a preliminary exploration. Future research should incorporate dynamic variables like rehabilitation details, complication management, and social support during this window to refine evidence-based, full-cycle functional management.

## Data Availability

The original contributions presented in the study are included in the article/Supplementary material, further inquiries can be directed to the corresponding author.
