# Supplementary material for: Predictors of functional deterioration from 90 days to 1 year after endovascular treatment for vertebrobasilar artery occlusion: a multicenter retrospective study
Source: Front Neurol. 2025 Nov 3;16:1671672. doi: 10.3389/fneur.2025.1671672 (PMC12621454; doi:10.3389/fneur.2025.1671672)
Supplement: Supplementary file 1 [file Table_1.DOCX]

Appendix S1

**Definition of variables:**

**PT (Procedure time)** was defined as the time interval from groin puncture to successful recanalization or the last failed attempt.

**DPT (Door-to-puncture time)** was defined as the time interval from patient arrival at the hospital to completion of groin puncture.

**No. of attempts** (The number of thrombectomy attempts) was generally defined as the number of operations performed on the occluded vessel using a thrombectomy device to achieve vascular recanalization.

**Imaging Analysis**All available imaging data underwent assessment by an independent core laboratory blinded to clinical outcomes and treatment allocation. Baseline pc-ASPECTS were determined using non-enhanced CT or MRI scans, applying the established scoring criteria. Within this system, individual brain regions receive a score of 1 for normal appearance or 0 for abnormality; consequently, higher total pc-ASPECTS values indicate less extensive infarction. The site of arterial occlusion was identified on initial vascular imaging (CT angiography, MR angiography, or digital subtraction angiography). Follow-up CT scans performed within 48 hours following endovascular therapy (EVT) were reviewed to detect intracranial hemorrhage. Two board-certified neuroradiologists independently interpreted each scan. Discrepancies between their assessments underwent adjudication by a third neuroradiologist, whose evaluation determined the final result.

**Strategies for Bias Mitigation**

**Minimizing Selection Bias:**

1. Predefined Eligibility Criteria: Strict participant inclusion and exclusion criteria were established prospectively to enhance the sample's representativeness of the broader target population.
2. Multicenter Design: Participant enrollment and data acquisition occurred across 65 national centers. This multicenter strategy enhances sample diversity and reduces geographic or site-specific selection bias.
3. Blinded Outcome Assessment: Independent assessors, masked to the treatment allocation, conducted the 3-month follow-up evaluations, including assessment of functional status using the modified Rankin Scale (mRS).

**Reducing Information Bias:**

1. Multi-Source Data Verification: Follow-up information accuracy and completeness were strengthened by cross-referencing data obtained from at least two distinct sources during patient tracking.
2. Centralized Imaging Review: An independent core imaging laboratory, blinded to treatment assignment, performed centralized review and re-evaluation of all baseline and follow-up neuroimaging studies.
3. Standardized Outcome Measurement: Internationally recognized and validated scales were employed for the uniform assessment of functional outcomes during follow-up visits, ensuring consistent data collection methodology.
4. Comprehensive Personnel Training: All staff involved in data gathering received thorough standardized training prior to the commencement of study activities to ensure protocol adherence.

Table S1. Results of multicollinearity analysis for each variable in Logistic regression analysis

| **variable** | Tolerance | VIF |
| --- | --- | --- |
| Sex | 0.938 | 1.066 |
| Age | 0.812 | 1.231 |
| Systolic blood pressure | 0.576 | 1.735 |
| Diastolic blood pressure | 0.580 | 1.723 |
| Admission Blood Glucose | 0.976 | 1.024 |
| Coronary heart disease | 0.931 | 1.075 |
| Stroke or TIA | 0.977 | 1.023 |
| Baseline NIHSS score | 0.974 | 1.027 |
| pc-ASPECTS | 0.957 | 1.045 |
| TOAST | 0.874 | 1.144 |
| PT | 0.936 | 1.069 |
| DPT | 0.932 | 1.073 |
| mTICI grade | 0.981 | 1.019 |
| No. of attempts | 0.907 | 1.103 |

Table S2. Hosmer-Lemeshow goodness-of-fit test in logistic regression analysis

| Steps | χ² | **P value** |
| --- | --- | --- |
| 1 | 13.455 | 0.097 |
| 2 | 3.016 | 0.933 |
| 3 | 7.118 | 0.524 |
| 4 | 10.970 | 0.203 |
| 5 | 5.907 | 0.658 |
| 6 | 3.368 | 0.909 |

Table S3. Hosmer-Lemeshow goodness-of-fit test in gender-stratified logistic regression analysis

| Steps | Female | | Male | |
| --- | --- | --- | --- | --- |
|  | χ² | **P value** | χ² | **P value** |
| 1 | 4.694 | 0.790 | 4.489 | 0.722 |
| 2 | 14.906 | 0.061 | 10.408 | 0.238 |
| 3 | 12.341 | 0.137 | 8.428 | 0.393 |
| 4 | 5.459 | 0.708 | 6.493 | 0.592 |

Table S4. Hosmer-Lemeshow goodness-of-fit test in age-stratified logistic regression analysis

| Steps | <70 years | | ≥70 years | |
| --- | --- | --- | --- | --- |
|  | χ² | **P value** | χ² | **P value** |
| 1 | 3.666 | 0.160 | <0.001 | 1.000 |
| 2 | 5.595 | 0.692 | 2.400 | 0.791 |
| 3 | 6.317 | 0.612 | 7.418 | 0.492 |
